# Mediterranean diet adherence on self-concept and anxiety as a function of weekly physical activity: an explanatory model in higher education

**DOI:** 10.3389/fnut.2023.1215359

**Published:** 2023-07-19

**Authors:** Eduardo Melguizo-Ibáñez, Gabriel González-Valero, Georgian Badicu, Fatma Hilal Yagin, José Manuel Alonso-Vargas, Luca Paolo Ardigò, Pilar Puertas-Molero

**Affiliations:** ^1^Department of Didactics of Musical, Plastic and Corporal Expression, Faculty of Education Sciences, University of Granada, Granada, Spain; ^2^Department of Physical Education and Special Motricity, Faculty of Physical Education and Mountain Sports, Transilvania University of Braşov, Braşov, Romania; ^3^Department of Biostatistics, and Medical Informatics, Faculty of Medicine, Inonu University, Malatya, Türkiye; ^4^Department of Teacher Education, NLA University College, Oslo, Norway

**Keywords:** Mediterranean diet (MD), self-concept, anxiety, physical self-concept, university students

## Abstract

**Introduction:**

Scientific literature has now demonstrated the benefits of an active lifestyle for people's psychological health. Based on the above statement, the aim was to (a) evaluate and adjust a structural equation model containing the variables anxiety, self-concept, and Mediterranean diet adherence and (b) contrast the proposed theoretical model by studying the differences between the variables according to the level of weekly physical activity in a sample of 558 university students.

**Methods:**

A non-experimental, exploratory, cross-sectional investigation has been proposed. Instruments such as the PREDIMED Questionnaire, the Beck Anxiety Inventory, the International Physical Activity Questionnaire, and the Form 5 Self-Concept Questionnaire were used to collect data.

**Results and discussion:**

The results illustrate that students showing low adherence to the Mediterranean diet had higher levels of anxiety (M = 0.95) than those showing a high degree of adherence (M = 0.75). It is also observed that young people with a high degree of adherence to the Mediterranean diet report higher scores in the different dimensions of self-concept compared to young people with a low degree of adherence. In conclusion, it is affirmed that young people who show a high degree of adherence to this dietary pattern show lower levels of anxiety and greater recognition of the different areas of their self-concept.

## 1. Introduction

The undergraduate educational stage is assumed to be a time period in which critical changes in dietary patterns (1, 2) and time spent in physical activity occur (3, 4). It has been observed that during this academic period, there is a decrease in the intake of healthy foods as well as less time devoted to physical exercise (5). Mainly, the intake of healthy foods is reduced due to the high academic load that young university students have (5), opting directly for precooked dishes that have a high caloric level (4, 5).

In terms of following and adhering to a healthy dietary pattern, numerous research studies have shown that the Mediterranean diet has health benefits for individuals (6). These benefits are not only due to the type of food but also to the quality, cooking, and nutrient supply of the food (7, 8). This dietary model is characterized by the ingestion of fresh, seasonal, and local products, as well as a diet low in animal fats and refined sugars (9). On the other hand, the consumption of fruits, vegetables, cereals, and legumes predominates higher consumption of oily fish, eggs, and foods rich in omega-3 fatty acids (6–9). Although there are many variations on this model, it is agreed that more than half of the micronutrient intake comes from carbohydrates (9). Regarding the quality of fats, it is observed that most of the fatty acids are monounsaturated (8, 9). Specifically, a low degree of adherence to the Mediterranean diet has been observed in the university population (5, 6). Studies have shown that positive adherence to the Mediterranean diet has health benefits for young people (10). Knight et al. (10) noted that positive follow-up helps increase the quality of life and reduces the risk of cardiovascular disease, as well as various types of cancer. Likewise, it has been observed that a positive follow-up to this dietary pattern, together with an adequate level of physical activity, provides benefits to the cognitive and emotional areas of young people (6).

Another element that provides numerous benefits for physical and mental health is the practice of regular physical activity (11). Physical activity is defined as any bodily movement performed by skeletal muscles, which involves significant energy expenditure (12). Currently, for the practice of physical activity to be beneficial, the World Health Organization (13) has established different time ranges depending on the age range. In terms of time, that organization (13) states that people between 18 and 64 years of age should perform between 150 and 300 min of moderate aerobic physical activity per week. Regarding the type of physical activity, the World Health Organization (13) states that if the physical activity is aerobic and vigorous, it should be between 75 and 150 min per week. In addition, if moderate aerobic physical activity exceeds 300 min and vigorous aerobic physical activity exceeds 150 min per week, additional mental health benefits are obtained (11). Being active from the physical point of view denotes improvements at the organic level, such as improved muscular and cardiorespiratory fitness, improved functional and bone health, reduced risk of hypertension, and prevention of different types of cancer (13). Regarding the psychological area, it has been observed that the regular practice of physical exercise brings benefits to the mental conception that the subject has of himself (14).

Self-concept is defined as the mental representation that a person creates of himself/herself as he/she interacts with and relates to the different environments of his/her daily life (15). Initially, this construct was conceived in a unidimensional way (16), but a multidisciplinary view of it has been constructed (17). The study model proposed by Shavelson et al. (18) differentiates self-concept into two subdimensions, namely, academic and non-academic. The latter is made up of physical, emotional, family, and social dimensions (18). This view has been studied in numerous investigations (19, 20), as it does not focus exclusively on academic elements but also on areas closely related to physical and mental wellbeing. One of the dimensions that gains more strength within the study of self-concept is the physical area (17). In this area, it has been observed that many young people show dissatisfaction with their physical condition (17). If this dissatisfaction is prolonged over time, disorders related to physical appearance may develop, directly affecting the emotional area (15).

Poor attention to mental health can lead to mental illnesses such as anxiety (21). This state is characterized by the association of symptoms such as muscle tension, a high degree of irritability, and a high state of worry (21). The study by Dasinger and Solmon (22) found that regular physical activity helps to reduce this state due to the release of neurotransmitters (4). Likewise, Marchena et al. (23) and Trigueros et al. (24) developed the idea that a healthy food intake and a positive adherence to a healthy diet have a positive effect on mental health, helping to prevent the occurrence of disturbing states. Likewise, it has been observed that young people who show poorer adherence to the Mediterranean diet and a sedentary lifestyle show higher levels of anxiety (24). It has been found that the university population shows high levels of anxiety about the academic environment (23). Despite this, many young people show a process of emotional overeating consisting of uncontrolled food intake to palliate the effects of anxiety (23, 24).

Once the problems that are evident in this study have been contextualized, this study aimed to (a) evaluate and fit a structural equation model containing the variables anxiety, self-concept, and Mediterranean diet adherence and (b) contrast the proposed theoretical model by studying the differences between the variables according to the level of weekly physical activity in a sample of 558 university students.

## 2. Material and methods

### 2.1. Sample and design

An exploratory, cross-sectional, and *ex post facto* study of 558 Spanish university students has been proposed. The sample is made up of university students in educational sciences. Focusing attention on the sociodemographic variables, the students' ages were between 18 and 31 years (25.09 ± 6.22). Regarding the distribution of the sample according to sex, three-quarters of the population (75%) belonged to the female sex, and one-quarter (25%) belonged to the male sex. Convenience sampling was employed to collect the data. Likewise, responses from participants who did not meet the inclusion criteria have been eliminated (studying a degree related to educational sciences).

### 2.2. Variables and instruments

The variables that make up the study and the instruments used for data collection are detailed below.

**Own elaboration Questionnaire**: It was used to collect age and sex (male/female). This instrument was used to add complementary data to the study sample.

**Beck Anxiety Inventory:** It has been used to collect the anxiety variable. It was initially settled by Beck et al. (25). According to the sample size of this research, the version adapted by Sanz and Navarro (26) has been used. The reason why the version of Sanz and Navarro (26) was used is because this version is adapted for university students. It consists of 21 items (For the past month I have felt unable to relax) that are answered through a 4-level Likert scale (0 = Not at all; 3 = Severely). This questionnaire has shown a high degree of reliability with a value of α = 0.939 in this study. It was decided to use this instrument because of its high degree of reliability. Likewise, this questionnaire offers an analysis of anxiety as studied in this research.

**Self-Concept Questionnaire Form 5:** It was designed and adapted to Spanish by García and Musitu (27). It consists of 30 items (I do my school work well) that are answered on a Likert scale. The reliability analysis showed in this research a value of α = 0.889. This instrument assesses self-concept under the theory developed by Shavelson et al. (18). This allows for a multidimensional view, wherein each self-concept variable can be studied as an independent variable.

**Predimed Questionnaire:** It was elaborated by Schöder et al. (28). The version by Álvarez-Álvarez et al. (29) was used due to the characteristics of the sample under study. The reason for using the version of Álvarez et al. (29) is due to the high degree of reliability and the adaptation of this instrument to the population under study. It consists of 14 items (e.g., Do you use olive oil as your main culinary fat? and How many sweet or carbonated beverages do you drink per day?) which are answered dichotomously and indicate consumption amounts. Once all the items had been answered and according to the final score, the responses were categorized into low, medium, and high adherence. The reliability analysis showed in this research a value of α = 0.830. This questionnaire has been used because it is a very reliable one to measure adherence to the Mediterranean diet. In addition, this version is the one proposed for the adult population.

**International Physical Activity Questionnaire:** It has been employed in the Spanish version adapted for university students (30). This questionnaire evaluates through time and frequency the type of physical activity performed in the last week (how many days did you do vigorous physical activities like heavy lifting, digging, aerobics, or fast bicycling in the last 7 days?). The responses are classified into three levels, namely, low, moderate, and high. The reliability analysis showed in this research a value of α = 0.815. This instrument was used for this study due to its high level of internal consistency. It is also a very useful questionnaire.

### 2.3. Procedure

The process related to the preparation of this study is described below. At an initial moment, once the initial idea was presented, a bibliographic search was carried out. This was carried out to determine the most reliable instruments to collect the data.

Once the design of the study was conceived at the University of Granada, specifically at the Faculty of Education Sciences, the questionnaire began to be sent to the different young people. The research was publicized through the different social networks of the Department of Didactics of Musical, Plastic, and Corporal Expression to try to contact as many students as possible. Before students were given access to the questionnaire, they were asked to participate on a voluntary basis. They were also assured that the data would be treated anonymously and exclusively for scientific purposes. Once this was done, students were given access to the questionnaire they had created. Due to the COVID-19 health crisis, to establish the least contact with the participants, the data were collected virtually. For this purpose, the questionnaire was registered on the Google Forms platform. Prior to creating this questionnaire, the research group agreed to duplicate two questions. The reason for this decision was to avoid registering participants who responded randomly. This resulted in the discarding of a total of 78 participants.

For the ethical aspects of the research, the ethical criteria registered in the Declaration of Helsinki were followed. In addition, to ensure greater ethical rigor, this study was continuously supervised by an ethics committee of the University of Granada (2966/CEIH/2022).

### 2.4. Statistical analysis

The IBM SPSS 25.0 statistical program was used for the comparative analysis. Initially, the homogeneity degree of the results was studied through the Kolmogorov-Smirnov test. The aforementioned test showed a normal distribution of the results. Subsequently, for the purpose of this research, a single-factor ANOVA was used. Cohen's standardized *d* (31) was used to study the effect size.

**Figure 1:**
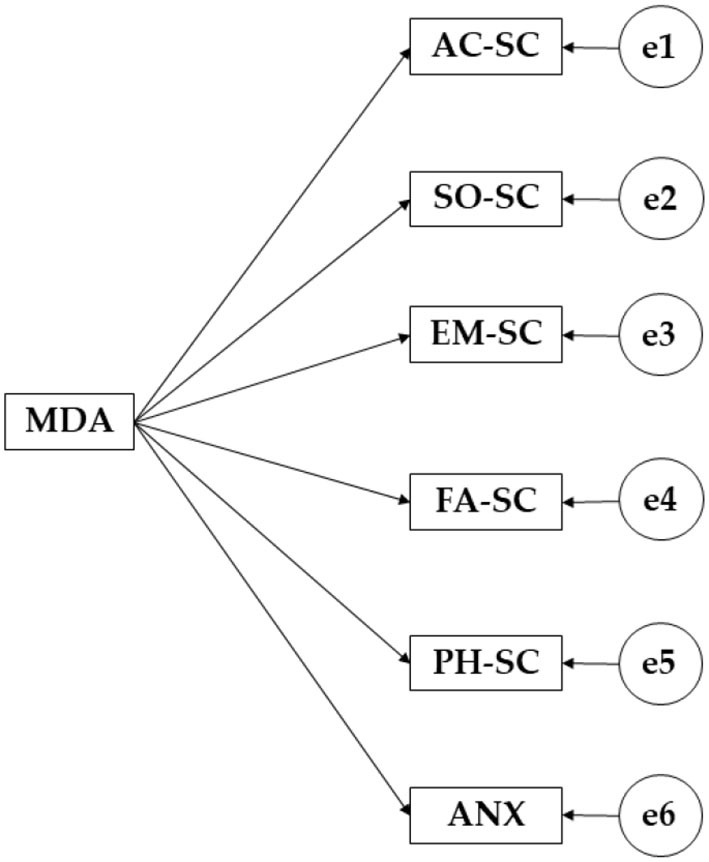
Research base model.

The IBM SPSS AMOS 26.0 software was employed to evaluate and adjust the structural equation models (Figure 1). The models are made up of endogenous and exogenous variables. The variable adherence to the Mediterranean diet serves as an exogenous variable, while all the dimensions of self-concept and anxiety act as endogenous variables. For endogenous variables, causal explanations have been carried out. These have been carried out based on the association between the indicator and the degree of measurement reliability. Hence, it has been possible to introduce the observation error. Likewise, the unidirectional arrows symbolize the regression weight between the latent and observed variables and are interpreted as regression weights. For the comparative and exploratory analyses, the significance level was established at a 95% confidence interval.

Based on the criteria established to adjust the equation models, attention will be focused on the values of the following indices (32, 33). The first one is related to the chi-square test, where non-significant values show a good fit (32). The comparative fit index, incremental reliability index, goodness of fit index, and root mean square approximation (33) will also be used. For the first three indices, the values must be higher than 0.900 (33). For the root mean square approximation, the value must be lower than 0.100 (32, 33). In addition to focusing attention on the size and susceptibility of the sample (34), attention should be paid to other adjustment indices such as the Tucker-Lewis index (34). For this index, the values must be >0.900 (34). Finally, with respect to the sampling error, a sampling error of 3.81% was obtained for a confidence level of 97%.

## 3. Results

Table 1 shows the comparative analysis of the data. For the anxiety variable, it was observed that participants who showed optimal adherence (M = 0.75) reflected lower levels of anxiety than those who showed low adherence (M = 0.95). Continuing with the self-concept, it is observed that young people who show a high degree of adherence to the Mediterranean diet obtain greater recognition in the academic, social, family, and physical areas (M = 4.06; M = 3.46; M = 3.49; M = 3.33) compared to those showing a low level of adherence (M = 3.62; M = 3.27; M = 3.34; M = 2.79; M = 1.97). Very different results are observed for the emotional self-concept, showing a greater recognition of participants who need to improve adherence to this dietary pattern (M = 3.10). For this dimension of self-concept, higher scores are observed for participants who show a low degree of adherence (M = 3.06) compared to those who show a high degree of adherence (M = 3.04). With regard to the practice of physical activity, higher levels were observed for participants who showed a high degree of adherence to the Mediterranean diet (M = 2.43).

**Table 1:** Comparative analysis of variables according to the level of Mediterranean diet adherence.

		**N**	**M**	**SD**	***F***	***P***	**ES (d)**	**95% CI**
ANX	High adherence	221	0.75	0.61	2.659	≥0.05	(–)	(–)
	Needs to improve	263	0.77	0.64				
	Low adherence	73	0.95	0.65				
AC	High adherence	221	4.06	0.65	12.058	≤ 0.05^a, b^	0.343^a^	[0.163; 0.523]^a^
	Needs to improve	263	3.81	0.79			0.648^b^	[0.378; 0.918]^b^
	Low adherence	73	3.62	0.76				
SO	High adherence	221	3.46	0.35	7.213	≤ 0.05^b^	0.543^b^	[0.275; 0.811]^b^
	Needs to improve	263	3.44	0.41				
	Low adherence	73	3.27	0.35				
EM	High adherence	221	3.04	0.80	0.364	≥0.05	(–)	(–)
	Needs to improve	263	3.10	0.78				
	Low adherence	73	3.06	0.88				
FA	High adherence	221	3.49	0.37	4.276	≤ 0.05^b^	0.397^b^	[0.131; 0.664]^b^
	Needs to improve	263	3.41	0.47				
	Low adherence	73	3.34	0.40				

a Differences between high adherence and needs to improve.

b Differences between high adherence and low adherence.

c Differences between needs to improve and low adherence.

N, Number of participants; M, mean value; SD, standard deviation; F, F-test; P, significance level; ES, effect size; 95% CI, 95% confidence interval; ANX, Anxiety; AC, academic self-concept; SO, social self-concept; EM, emotional self-concept; FA, family self-concept; PH, physical self-concept; PA, physical activity.

The model presented for the participants who showed a low physical activity level evidenced a good fit. A non-significant value was obtained (X^2^ = 51.612; df = 15; pl = 0.000) for the chi-square test. The values obtained for the fit indices are shown below. The Comparative Fit Index (CFI), the Normalized Fit Index (NFI), the Incremental Fit Index (IFI), and the Tucker-Lewis Index (TLI) obtained values of 0.968, 0.961, 0.969, and 0.929 for each one. The root mean square error of approximation analysis (RMSEA) was 0.025.

**Figure 2:**
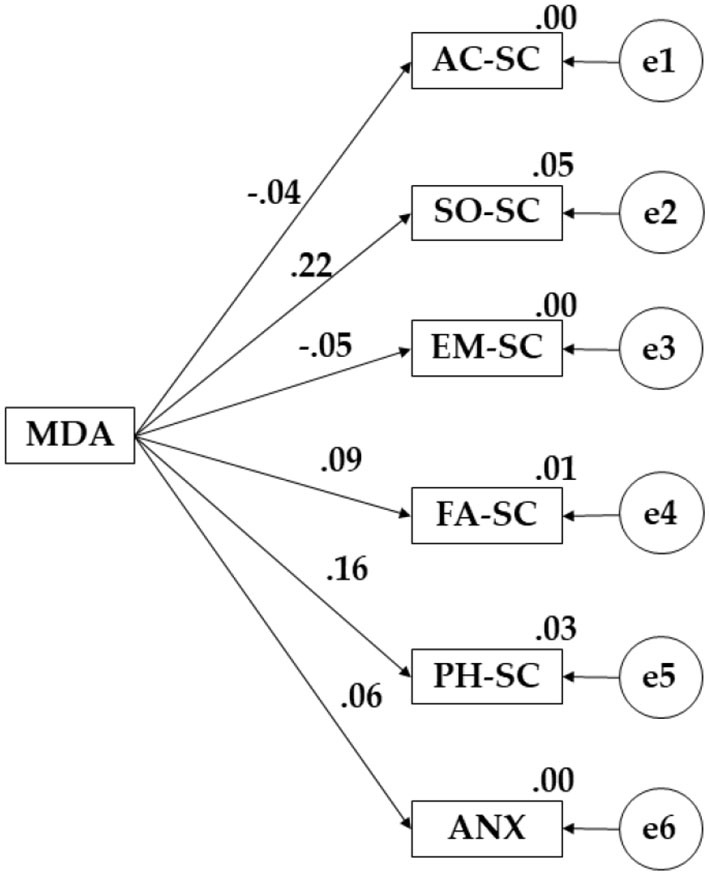
Proposed model for low-level PA.

Figure 2 and Table 2 evidence the regression weights of the proposed theoretical model for participants showing a low level of physical activity. Mediterranean diet adherence was positively associated with social self-concept (*p* < 0.05; β = 0.217), emotional self-concept (β = 0.048), family self-concept (β = 0.093), physical domain (β = 0.161), and anxiety (β = 0.498). A negative effect was observed in the academic area (β = −0.041).

**Table 2:** Standardized regression weights obtained for participants with a low level of PA.

**Effect direction**	**RE.WE**				**ST.RE.WE**
	**EST**	**ES.ERR**	**CR.RA**	***P***	**EST**
AC ←MDA	−0.272	0.587	−0.464	0.643	−0.041
SO ←MDA	0.728	0.291	2.503	< 0.05	0.217
EM ←MDA	0.311	0.570	0.545	0.586	0.048
FA ←MDA	0.308	0.294	1.049	0.294	0.093
PH ←MDA	0.875	0.477	1.836	0.066	0.161
ANX ←MDA	0.322	0.476	0.677	0.498	0.060

ANX, Anxiety; AC, academic self-concept; SO, social self-concept; EM, emotional self-concept; FA, family self-concept; PH, physical self-concept; PA, physical activity; MDA, Mediterranean diet adherence; RE.WE, Regression weights; ST.RE.WE, standardized regression weights; ES.ERR, estimation error; CR.RA, critical ratio; EST, estimation.

The structural equation model presented for the students who evidenced a moderate level of physical activity was a good fit. A non-significant value was obtained (X^2^ = 55.289; df = 15; *p*l = 0.000) for the chi-square test. The values obtained for the fit indices are shown below. The Comparative Fit Index (CFI), the Normalized Fit Index (NFI), the Incremental Fit Index (IFI), and the Tucker-Lewis index (TLI) obtained values of 0.924, 0.961, 0.918, and 0.905, respectively. The value obtained by root mean square error of approximation analysis (RMSEA) was 0.030.

**Figure 3:**
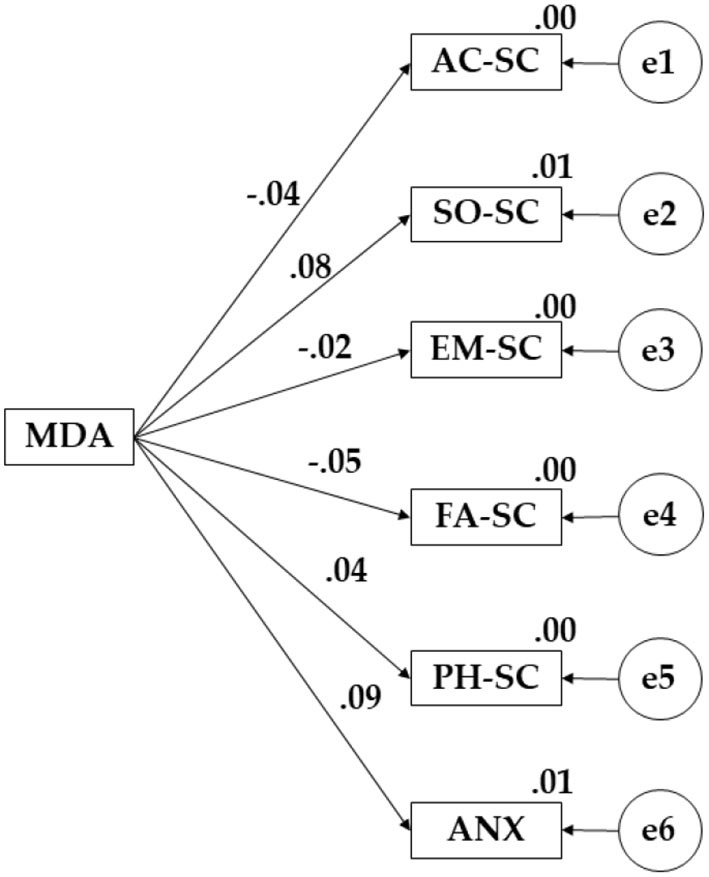
Proposed model for moderate levels of PA.

Figure 3 and Table 3 evidence the regression weights of the proposed theoretical model. Mediterranean diet adherence was positively associated with social self-concept (β = 0.082), physical self-concept (β = 0.045), and anxiety (β = 0.085). In contrast, negative relationships were observed with academic domain (β = −0.036), emotional area (β = −0.020), and family self-concept (β = 0.047).

**Table 3:** Standardized regression weights obtained for participants with a moderate level of PA.

**Effect direction**	**RE.WE**				**ST.RE.WE**
	**EST**	**ES.ERR**	**CR.RA**	***P***	**EST**
AC ←MDA	−0.224	0.473	−0.473	0.636	−0.036
SO ←MDA	0.237	0.221	1.072	0.284	0.082
EM ←MDA	−0.127	0.481	−0.265	0.791	−0.020
FA ←MDA	−0.163	0.267	−0.609	0.542	−0.047
PH ←MDA	0.240	0.413	0.581	0.561	0.045
ANX ←MDA	0.388	0.350	1.110	0.267	0.085

ANX, Anxiety; AC, academic self-concept; SO, social self-concept; EM, emotional self-concept; FA, family self-concept; PH, physical self-concept; PA, physical activity; MDA, Mediterranean diet adherence; RE.WE, Regression weights; ST.RE.WE, standardized regression weights; ES.ERR, estimation error; CR.RA, critical ratio; EST, estimation.

The structural equation model presented for the participants who showed a high level of physical activity evidenced a good fit. A non-significant value was obtained (X^2^ = 54.892; df = 15; pl = 0.000) for the chi-square test. The values obtained for the fit indices are shown below. The Comparative Fit Index (CFI), the Normalized Fit Index (NFI), the Incremental Fit Index (IFI), and the Tucker-Lewis Index (TLI) obtained values of 0.947, 0.958, 0.950, and 0.910, respectively. On the contrary, the value obtained by root mean square error of approximation analysis (RMSEA) was 0.040.

**Figure 4:**
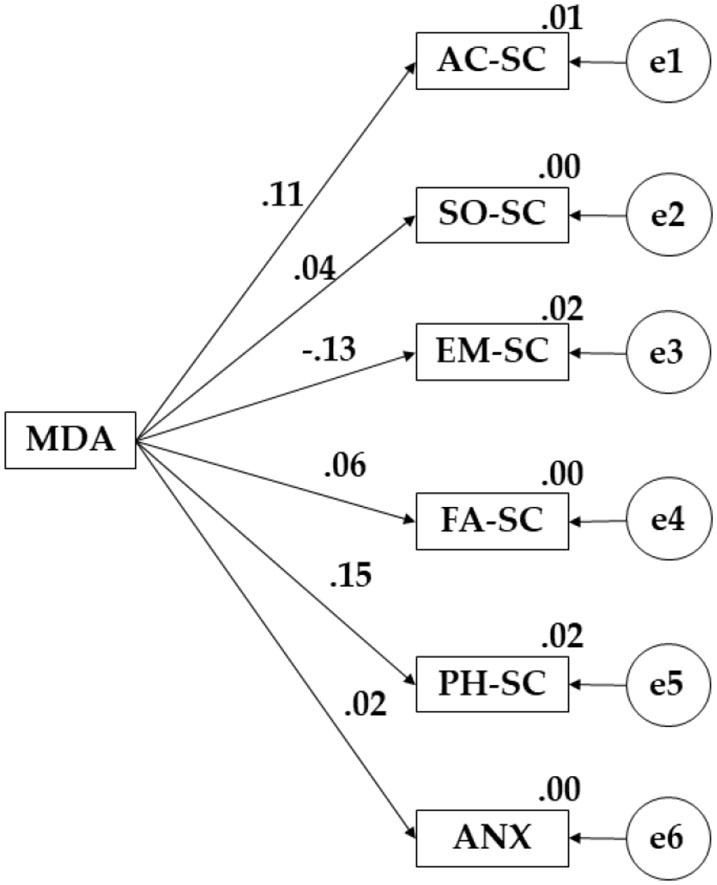
Proposed model for high-level PA.

It is evidenced in Table 4 and Figure 4 that the Mediterranean diet has a positive effect on academic self-concept (β = 0.109), social self-concept (β = 0.038), family self-concept (β = 0.064), physical self-concept (β = 0.146), and anxiety (β = 0.024). On the contrary, Mediterranean diet adherence was negatively associated with emotional self-concept (β = −0.132).

**Table 4:** Standardized regression weights obtained for the participants with a high level of PA.

**Effect direction**	**RE.WE**				**ST.RE.WE**
	**EST**	**ES.ERR**	**CR.RA**	***P***	**EST**
AC ←MDA	0.688	0.392	1.757	0.079	0.109
SO ←MDA	0.136	0.223	0.611	0.541	0.038
EM ←MDA	−0.994	0.462	−2.150	0.032	−0.132
FA ←DA	0.258	0.249	1.037	0.300	0.064
PH ←MDA	1.119	0.471	2.373	< 0.05	0.146
ANX ←MDA	0.143	0.367	0.389	0.697	0.024

ANX, Anxiety; AC, academic self-concept; SO, social self-concept; EM, emotional self-concept; FA, family self-concept; PH, physical self-concept; PA, physical activity; MDA, Mediterranean diet adherence; RE.WE, Regression weights; ST.RE.WE, Standardized regression weights; ES.ERR, estimation error; CR.RA, critical ratio; EST, estimation.

The previous tables (Tables 2–4) show the differences found according to the models proposed. Considering the relationship between adherence to the Mediterranean diet and self-concept, a greater effect was observed for participants with a high level of physical activity (β = 0.109). Higher scores are observed in the relationship between adherence to the Mediterranean diet and social self-concept (β = 0.217), adherence to the Mediterranean diet and emotional self-concept (β = 0.048), adherence to the Mediterranean diet and family self-concept (β = 0.093), and between adherence to the Mediterranean diet and the practice of physical activity (β = 0.161) for participants who show a low level of physical activity. Finally, participants showing a high level of physical activity evidenced a better relationship between adherence to the Mediterranean diet and anxiety (β = 0.024).

## 4. Discussion

Once the relationship between the variables has been analyzed, the Discussion Section aims to compare the results found with those of other studies similar to this one. In this case, a comparative and exploratory analysis of the variables, namely anxiety, adherence to the Mediterranean diet, self-concept, and physical activity practice has been presented. The exploratory analysis was presented using a multigroup structural equation model.

The comparative analysis shows that young people who show a high degree of adherence to the Mediterranean diet have lower levels of anxiety. The study by Marchena et al. (23) conducted in the university population found a negative relationship between anxiety and adherence to the Mediterranean diet. This research (23) also found that low adherence to the Mediterranean diet is associated with emotional problems and higher levels of negative emotions such as anxiety and stress. Given the appearance of these negative states in the university population, the studies by Marchena et al. (23) and Trigueros et al. (24) found positive relationships between eating behavior problems and the appearance of anxiety or stress.

This analysis then shows that a high degree of adherence to the Mediterranean diet has a beneficial effect on the different dimensions of self-concept. In view of these findings, the research carried out by Melguizo-Ibáñez et al. (35) on a population of university students of educational sciences found that the Mediterranean diet acts beneficially on all dimensions of self-concept. In line with the previous study, Ubago-Jiménez et al. (5) found in the university population benefits of a positive adherence to the Mediterranean diet, such as a better conception of fitness and notable improvements in the dimensions of interpersonal intelligence, which is positively related to self-concept. Similarly, the study conducted by Marchena et al. (23) also found that university students with a high adherence to the Mediterranean diet show a better conception of all dimensions of self-concept and self-esteem. The Mediterranean diet has been shown to be emotionally, socially, and physically beneficial due to the types of foods that make up this dietary pattern (5, 23, 35).

Likewise, the comparative analysis illustrates that participants who show high adherence to the Mediterranean diet obtain a higher level of physical activity. The research carried out by López-Gil et al. (36) found that from the early ages of human development, a positive link should be created toward an active and healthy lifestyle. Likewise, in the adult population, an increase in the practice of physical activity has been found, as well as a greater consumption of foods present in the Mediterranean diet (37). In view of the above statement, it has been found that a healthy diet together with an active lifestyle helps to increase the life expectancy of young women and to prevent cardiorespiratory and cardiovascular diseases (37).

Next, the exploratory analysis reveals the associations between adherence to the Mediterranean diet and the various dimensions of self-concept based on the type of physical activity performed.

Regarding the relationship between academic self-concept and adherence to the Mediterranean diet, it was found that greater exercise intensity improved the relationship between these variables. In this case, the research developed by Melguizo-Ibáñez et al. (35) in a sample of students of educational sciences found a significant improvement between a higher intensity of physical activity and academic self-concept. In view of these findings, the research carried out by López-Gil et al. (36) found that the practice of physical activity at a moderate or vigorous level reports improvements in executive functions, improving attention and concentration.

Continuing with the results of the relationship between adherence to the Mediterranean diet and social self-concept, a better relationship is observed for lighter physical activity. Research conducted by Laiou et al. (37) in an adult population concluded that the practice of any type of physical activity helps promote socialization among people. Similarly, Zhang et al. (38) found that being physically active helps to have a better social image as well as increase the degree of socialization among peers. Regarding the type of physical activity, it has been found that low-intensity sports practice allows a higher degree of interaction among peers (37, 38).

Regarding the emotional domain and adherence to the Mediterranean diet, a worsening of both variables was obtained as the intensity of physical exercise increased. The study conducted by Melguizo-Ibáñez et al. (39) in an adolescent population found that in the academic stages prior to university, positive relationships are found between the emotional, physical, and health domains. Furthermore, this research (39) found that the development of these patterns in the early stages of development favors the persistence of these behaviors into adulthood. Likewise, González-Valero et al. (40) found that within the university student environment, the practice of physical exercise helps to improve attention, clarity, and emotional repair. In addition, Trigueros et al. (41) found that positive adherence to a healthy dietary pattern contributes to improving the cognitive sphere, which is directly affected by emotions.

Focusing on the family sphere of self-concept, it was found that university students who practice physical activity at a lower intensity reflect better results in the family sphere and adherence to a healthy dietary pattern. In view of these findings, Melguizo-Ibáñez et al. (42) found that in the early stages of adolescent development, the family environment plays a key role in the creation of an active and healthy lifestyle. Likewise, this research (42) affirms that families with a low socioeconomic level show less concern for the development of healthy and active behaviors in their infants. Within the university setting, Marco-García et al. (43) found that young people acquire a greater degree of dependence and therefore show a detachment from the nuclear family. Likewise, the development of active behavior may be affected by the academic environment (43).

Regarding the physical dimension of self-concept, it was found that participants with a low level of physical activity showed a better effect of the Mediterranean diet in this area. Given such findings, Melguizo-Ibáñez et al. (44) found that in adolescents and young adults, there is a greater concern for the care of body appearance. Likewise, the study carried out by Fernández-Bustos et al. (45) found a higher level of self-concept for young people who show a moderate or vigorous level of physical activity.

Finally, it is observed that for the relationship between adherence to the Mediterranean diet and anxiety, participants who practice vigorous physical activity obtain better results. Similar results were obtained by Marchena et al. (23), whereas Jayo-Montoya et al. (46) affirm that the practice of physical activity together with a healthy dietary pattern helps to reduce the disruptive states generated during daily life due to the segregation of neurotransmitters.

### 4.1. Limitations and future perspectives

Once the objectives of this research have been answered, it is necessary to mention its limitations. The first of these is related to the study population. Despite having obtained a significant sample, generalizations cannot be made. This is mainly because the sample is not significant at the national or regional level. The next aspect related to the sample is the degree of homogeneity of the sample. The sample for this study is made up mostly of female participants. Another limitation would also be the research design since only one data collection can be interpreted at the time the data were collected. This means that the data should be interpreted with caution. Considering the prospects, this research can be used as a starting point to develop an intervention program with a longitudinal design.

Several practical applications have emerged from this research. The first of these is related to the physical and nutritional training that students receive. It would be necessary that during the last stages of Compulsory Secondary Education, students receive information related to the benefits of an active and healthy lifestyle. Likewise, this research can be considered a pilot study that will shed light on an intervention program for the training of future teachers. Another possible practical application would be the need to carry out a greater number of intervention programs aimed at improving the health status of young people.

## 5. Conclusion

The comparative analysis shows that young people who demonstrate a high degree of adherence to the Mediterranean diet show lower levels of anxiety. It is also observed that a high degree of adherence to the Mediterranean diet brings benefits in the family, academic, social, and physical dimensions of self-concept. In addition, participants with a high degree of adherence show a greater level of physical activity.

The exploratory analysis shows that a high level of weekly physical activity shows a better effect of the Mediterranean diet on the academic areas of self-concept and anxiety. Young people who practice a low level of weekly physical exercise show a better effect of the Mediterranean diet on the social and family dimensions. Finally, negative effects of the Mediterranean diet on emotional, family, and academic areas of self-concept were observed.

Finally, this study concludes that an active lifestyle demonstrates improvements in the effect of a healthy dietary pattern on the different dimensions of self-concept and anxiety.

## Data availability statement

The raw data supporting the conclusions of this article will be made available by the authors, without undue reservation.

## Ethics statement

The studies involving human participants were reviewed and approved by Ethics Committee 2966/CEIH/2022 of the University of Granada approved the present research. Written informed consent to participate in this study was provided by the participants' legal guardian/next of kin.

## Author contributions

EM-I, LPA, JA-V, PP-M, and GB conceived and designed the experiments. EM-I, GG-V, JA-V, PP-M, GB, and FHY performed the experiments and wrote the article. EM-I, GG-V, JA-V, PP-M, GB, and LPA analyzed and interpreted the data. All authors contributed to the article and approved the submitted version.
